# pH-Sensitive oligopeptide magnetic mesoporous silica beads for deoxyribonucleic acid extraction†

**DOI:** 10.1039/d4na00987h

**Published:** 2025-01-21

**Authors:** Sihua Qian, Yiting Wang, Junjie Fan, Tong Kong, Yuhui Wang, Kaizhe Wang, Yufeng Liao, Li Wang, Jianping Zheng

**Affiliations:** a College of Chemistry, Jilin Normal University Siping 136000 P. R. China liwang@jlnu.edu.cn; b Ningbo Cixi Institute of Biomedical Engineering, Laboratory of Advanced Theranostic Materials and Technology, Ningbo Institute of Materials Technology and Engineering, Chinese Academy of Sciences Ningbo 315300 P. R. China zhengjianping@nimte.ac.cn; c Department of Clinical Laboratory Ningbo No. 2 Hospital Ningbo 315010 P.R. China liaoyufengs@163.com

## Abstract

Exploring novel synthesis strategies for magnetic beads to extract nucleic acids is of great significance in the field of *in vitro* diagnostics. In the present research, monodisperse magnetic mesoporous silica beads were synthesized *via* the thermolysis reaction of Fe(acac)_3_ by using large-pore dendritic silica colloids as templates, and were further functionalized with a highly pH-sensitive histidine-glutamate co-oligopeptide for deoxyribonucleic acid extraction. The large-pore dendritic silica colloid scaffolds were utilized for high-density incorporation of superparamagnetic iron oxide nanoparticles within the vertical channels. The morphology and properties of the as-prepared pH-sensitive oligopeptide magnetic mesoporous silica beads were evaluated by transmission electron microscopy, scanning electron microscopy, vibrating sample magnetometry, X-ray photoelectron spectroscopy, X-ray diffraction testing and so on. The average size of the obtained magnetic beads was 370 nm in diameter with a narrow size distribution. The saturation magnetization and magnetic content of the resultant magnetic beads were 25 emu g^−1^ and 59%, respectively. Moreover, the magnetic mesoporous silica beads exhibited an obvious pH-responsive behavior. Due to these remarkable features, successful deoxyribonucleic acid capture using the as-prepared pH-sensitive oligopeptide magnetic mesoporous silica beads was achieved.

## Abstract 2

pH-Sensitive oligopeptide magnetic mesoporous silica beads were successfully prepared by using large-pore dendritic silica colloids as templates for DNA extraction.

## Graphical abstract

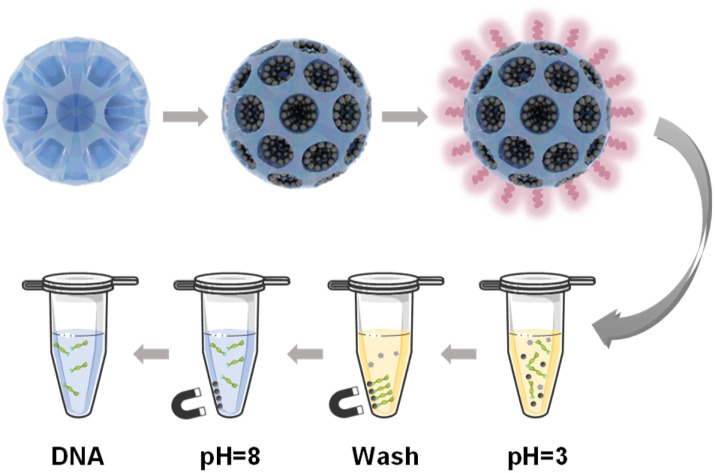

## Introduction

Deoxyribonucleic acid (DNA) extraction is an essential process in molecular biology and a fundamental step for subsequent sequencing, amplification, and biodetection.^1–8^ The traditional method for isolation of DNA based on phenol/chloroform extraction suffers from several drawbacks, including using highly toxic solvents, being labor- and time-consuming, and being unsuitable for processing of trace samples.^9–11^ Nowadays, employing various solid-phase supports for DNA extraction has become more and more attractive,^12–16^ among which magnetic beads are preferred due to their easy manipulation and low cost.^17–22^ Magnetic DNA purification is a clear improvement upon centrifuge-dependent isolation techniques when semi-automatic or fully automatic systems are considered.^23–26^ With the assistance of an external magnetic field, magnetic beads as solid-phase adsorbents can be removed readily.

Various methods have been developed for the preparation of magnetic beads,^27–31^ among which Ugelstad's activated swelling method is the most successful route and has formed a series of commercial products, Dynabeads.^32–36^ The preparation process of the activated swelling method generally involves the fabrication of monodisperse macroporous polymeric beads using seed polymerization technology. Subsequently, the internal and external surfaces of the macroporous beads are modified with –SO_3_ or –NO_2_ to impart hydrophilic properties to them. The beads are then immersed in an aqueous solution of iron salt, leading to the generation of superparamagnetic Fe_3_O_4_ or γ-Fe_3_O_4_ within the pores under suitable reaction conditions. Finally, a monomer containing active functional groups is chosen for swelling, polymerization, and coating of the beads to seal off pores and functionalize their surfaces.^37^ To meet the requirements of rapid isolation, magnetic beads produced by swelling methods have a micron-level size with a diameter typically ranging from 1 to 100 μm to contain enough magnetic components.^33,38^ However, micron-sized magnetic beads exhibit poor suspension and quick sedimentation, which are unfavorable for DNA extraction.

Compared with organic polymeric materials, inorganic ones possess the advantages of low toxicity, special structures, stable physicochemical properties and so on.^39,40^ Among inorganic substrates, mesoporous silica materials have been a hot topic due to their larger surface area and specific mesoporous channels, which can dramatically increase the binding capacity of target molecules onto the surface and pores.^41–45^ Furthermore, the integration of magnetic nanoparticles with mesoporous silica materials has attracted considerable attention in recent years owing to their excellent properties.^46–51^ Magnetic mesoporous silica nanocomposites are generally core–shell-type, which would reduce the magnetic response of the pure magnetic nanoparticles. Therefore, an alternative magnetic nanoparticles-mesoporous silica material integration type needs to be urgently developed.

**Fig. 1:**
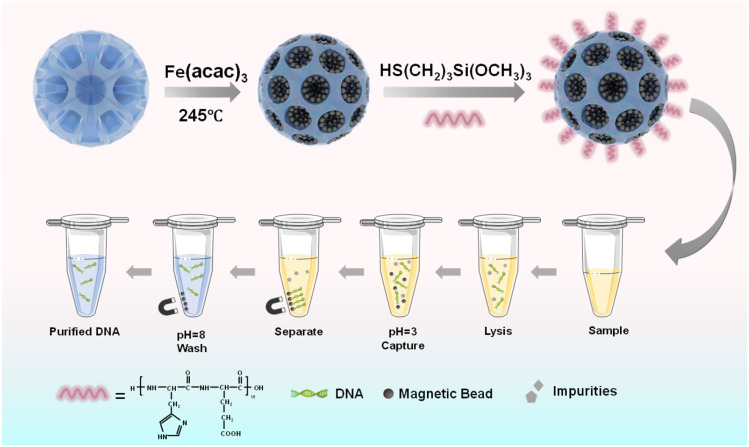
Schematic illustrations of the preparation process of pH-sensitive oligopeptide magnetic mesoporous silica beads and their application in deoxyribonucleic acid extraction.

Herein, to increase the suspension and magnetic response of magnetic beads, magnetic mesoporous silica beads with several hundreds of nanometers were prepared, and were further functionalized with a highly pH-sensitive histidine-glutamate (HE) co-oligopeptide for DNA extraction, as illustrated in Fig. 1. We first prepared dendritic silica colloids (dSiO_2_) with uniform size and expected monodispersity through an anion-assisted approach. Then, the Fe_3_O_4_@dSiO_2_ composite was obtained *via* the thermolysis reaction of Fe(acac)_3_ by using large-pore dSiO_2_ as templates, and the ultra-large pore channels of dSiO_2_ allowed the complete confinement of superparamagnetic Fe_3_O_4_ nanoparticles in the interior of the supports. Finally, the pH-responsive Fe_3_O_4_@dSiO_2_-(HE)_10_ composite was acquired *via* conjugate addition reactions between thiol functional groups and maleimide moieties. Oligopeptides have been particularly widely used to provide physical and biological stabilization to magnetic nanoparticles.^52^ The structures, properties, and surface morphologies of dSiO_2_, Fe_3_O_4_@dSiO_2_ and Fe_3_O_4_@dSiO_2_-(HE)_10_ were investigated by transmission electron microscopy, scanning electron microscopy, dynamic light scattering, inductively coupled plasma (ICP, magnetic content 59%) spectrometry, and vibrating sample magnetometry, respectively. Meanwhile, DNA separation experiments were carried out with these particles to evaluate their adsorption ability.

## Results and discussion

### Characterization of Fe_3_O_4_@dSiO_2_-(HE)_10_

**Fig. 2:**
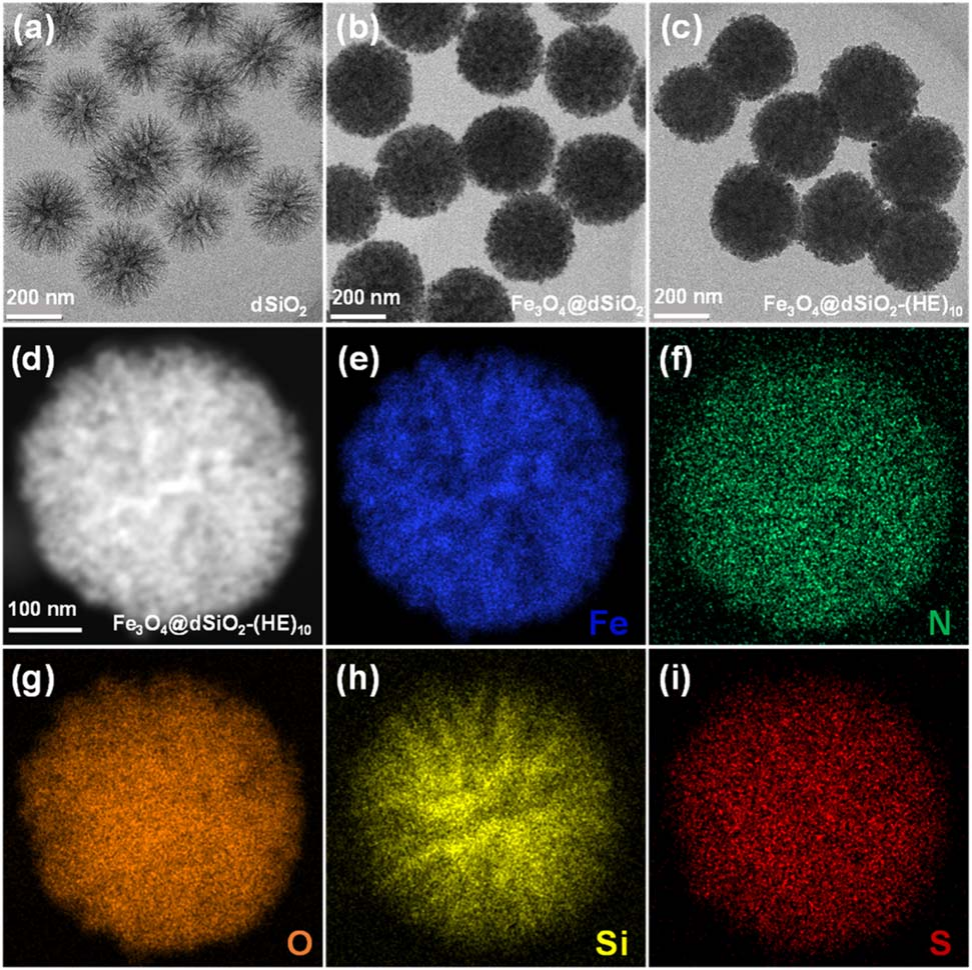
TEM images of dSiO_2_ templates (a), Fe_3_O_4_@dSiO_2_ (b), and Fe_3_O_4_@dSiO_2_-(HE)_10_ (c). STEM image (d) and EDS elemental (Fe, N, O, Si, and S) mapping images (e–i) of a single Fe_3_O_4_@dSiO_2_-(HE)_10_ nanosphere.

**Fig. 3:**
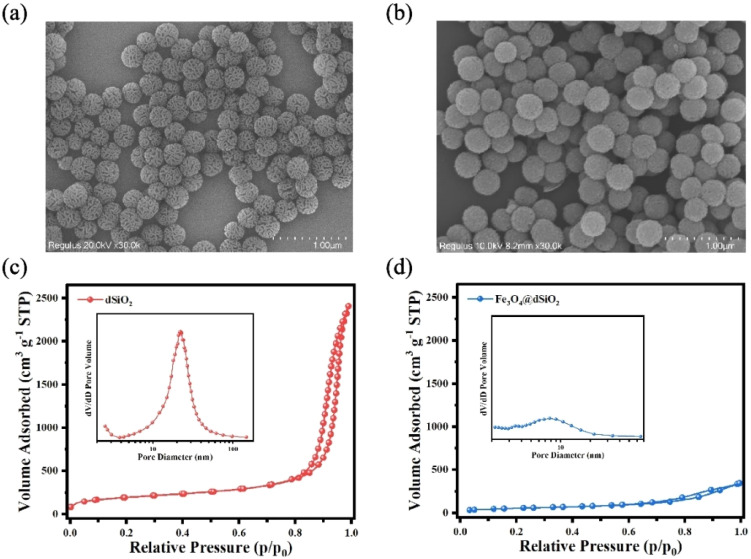
SEM images of dSiO_2_ templates (a) and Fe_3_O_4_@dSiO_2_ (b); nitrogen adsorption–desorption isotherms and the BJH pore size distributions (inset) of dSiO_2_ (c) and Fe_3_O_4_@dSiO_2_ (d).

The dSiO_2_ templates were synthesized using an anion-assisted approach, using cationic surfactant CTAB and NaSal as structure directing agents, TEA as a catalyst and TEOS as a silica source. In the TEM image of dSiO_2_, shown in Fig. 2a, the central-radial pores can be clearly seen. The SEM image of dSiO_2_ revealed an average pore-width of 20 nm at the external surface (Fig. 3a), and the large pore channels presented in the SEM image indicated the high accessibility of dSiO_2_'s inner surface for Fe_3_O_4_ immobilization. Additionally, the dSiO_2_ particles were monodisperse and highly uniform in size. The Fe_3_O_4_ nanoparticles were synthesized by the thermolysis reaction of Fe(acac)_3_ in 2-pyrrolidone at 245 °C. As shown in Fig. S1,† the diameter of Fe_3_O_4_ nanoparticles was 5–10 nm, and 2-pyrrolidone was chosen as both a stabilizer and solvent. To obtain a strong magnetic response and uniform magnetic nanoparticles, the dSiO_2_ that can serve as substrates for Fe_3_O_4_ deposition was added before the thermal decomposition of the iron precursor. As presented in Fig. 2b and 3b, all the radial channels of dSiO_2_ were occupied by a dense Fe_3_O_4_ layer, and the high coverage of small sized Fe_3_O_4_ confirmed a strong magnetic response and maintained the superparamagnetism of the Fe_3_O_4_@dSiO_2_ composites. After modification with the pH-sensitive histidine-glutamate co-oligopeptide *via* conjugate addition reactions between thiol functional groups and maleimide moieties, the morphology of Fe_3_O_4_@dSiO_2_-(HE)_10_ was similar to that of Fe_3_O_4_@dSiO_2_ (Fig. 2c). The distribution of hydrated particle sizes of dSiO_2_, Fe_3_O_4_@dSiO_2_ and Fe_3_O_4_@dSiO_2_-(HE)_10_ in an aqueous medium was also characterized, as presented in Fig. S2.† It can be found in Fig. S2† that the particle sizes of dSiO_2_, Fe_3_O_4_@dSiO_2_ and Fe_3_O_4_@dSiO_2_-(HE)_10_ in the aqueous medium satisfied the normal distribution and that the particle size distribution was narrow and symmetric, indicating there was little presence of either very small particles or very large ones. Most of the particles of dSiO_2_, Fe_3_O_4_@dSiO_2_ and Fe_3_O_4_@dSiO_2_-(HE)_10_ were distributed around the average hydrated particle sizes of 340 nm, 370 nm and 370 nm, respectively. The scanning transmission electron microscopy (STEM) image of Fe_3_O_4_@dSiO_2_-(HE)_10_ illustrated the even and compact distribution of Fe_3_O_4_ confined in the silica matrix (Fig. 2d). The energy-dispersive X-ray spectroscopy (EDS) mapping of Fe_3_O_4_@dSiO_2_-(HE)_10_ (Fig. 2e–i) confirmed the constituent elements of Fe_3_O_4_ (Fe), dSiO_2_ (Si and O), and histidine-glutamate co-oligopeptide modification (N and S).

The nitrogen adsorption–desorption isotherms reflected the evolution of the pore structure during the synthesis of Fe_3_O_4_@dSiO_2_ composites, as shown in Fig. 3c and d. They all exhibited typical IV-type curves with a hysteresis loop of the H2-type, which proved that the mesoporous structure of dSiO_2_ was not altered after loading Fe_3_O_4_. The dSiO_2_ templates exhibited large Brunauer–Emmett–Teller (BET) surface area (678.68 m^2^ g^−1^) and total pore volume (13.74 cm^3^ g^−1^). After loading Fe_3_O_4_, the BET surface area and total pore volume of dSiO_2_ decreased remarkably to 206.24 m^2^ g^−1^ and 0.53 cm^3^ g^−1^ due to the blockage of loaded Fe_3_O_4_ in the pore channels of dSiO_2_, which agreed well with the smooth spherical morphology. The Barrett–Joyner–Halenda (BJH) pore size distributions of the microspheres also indicated a shrinking of the pore size from 23.15 nm (inset in Fig. 3c) to 9.25 nm (inset in Fig. 3d), which was consistent with the TEM observations.

**Fig. 4:**
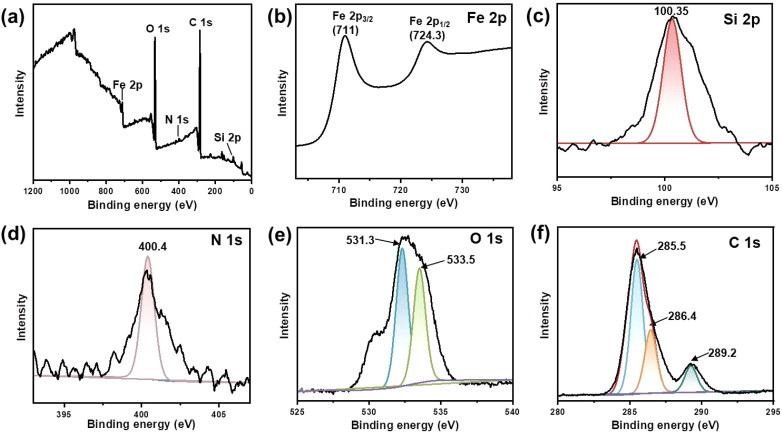
XPS spectra of Fe_3_O_4_@dSiO_2_-(HE)_10_. (a) Survey spectrum, (b) Fe 2p spectrum, (c) Si 2p spectrum, (d) N 1s spectrum, (e) O 1s spectrum, and (f) C 1s spectrum.

X-ray photoelectron spectroscopy (XPS) was utilized to analyse the elemental composition and chemical valence changes of Fe_3_O_4_@dSiO_2_-(HE)_10_. As shown in Fig. 4a, peaks at 711, 532.3, 400.4, 285.5 and 100.35 eV of the prepared Fe_3_O_4_@dSiO_2_-(HE)_10_ were assigned to binding energies of Fe 2p, O 1s, N 1s, C 1s and Si 2p, respectively, which indicated that the sample was composed of the elements Fe, O, N, C, and Si. The spectra for Fe 2p in the Fe_3_O_4_@dSiO_2_-(HE)_10_ nanoparticles exhibited peaks at 711 eV and 724.3 eV for Fe 2p_3/2_ and Fe 2p_1/2_, respectively (Fig. 4b), which revealed that Fe_3_O_4_ was successfully prepared. The spectra for Si 2p exhibited a Si peak at 100.35 eV, which corresponded to Si–O, as described in Fig. 4c. Since the oligopeptide contained numerous –NH_2_ groups, the peak for the amino group confirmed the successful modification of the oligopeptide. The spectra for N 1s had a peak at 400.4 eV (Fig. 4d), which corresponded to the –NH_2_ group, demonstrating the presence of amino groups in nanoparticles. The O 1s binding energies at 531.3 eV and 533.5 eV were attributed to the lattice oxygen in Fe_3_O_4_ and Si–O bonds in SiO_2_, respectively, as presented in Fig. 4e. As shown in Fig. 4f, the C 1s peak can be deconvoluted into three peaks at 285.5, 256.4 and 289.2 eV, corresponding to C=C, C–N and C=O, respectively. The XPS results also confirmed the successful formation of Fe_3_O_4_@dSiO_2_-(HE)_10_.

**Fig. 5:**
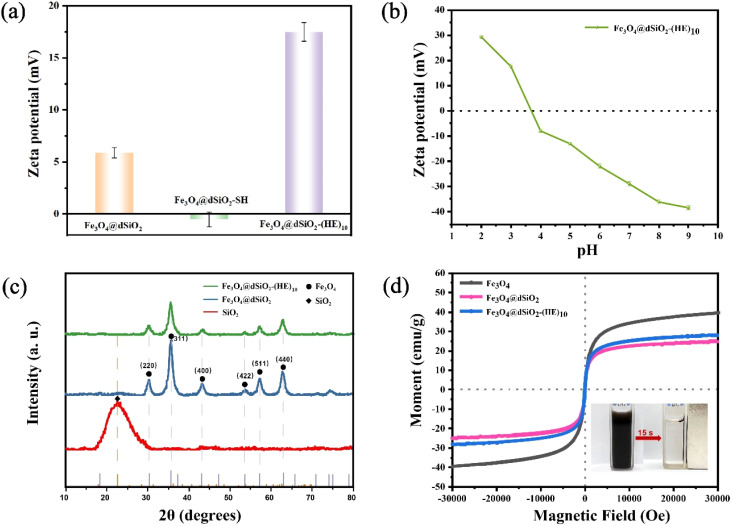
(a) Zeta potentials of Fe_3_O_4_@dSiO_2_, Fe_3_O_4_@dSiO_2_-SH and Fe_3_O_4_@dSiO_2_-(HE)_10_ at pH = 3.0; (b) zeta potentials of Fe_3_O_4_@dSiO_2_-(HE)_10_ in different pH solutions, and pH 3.75 turned out to be the isoelectric point of Fe_3_O_4_@dSiO_2_-(HE)_10_; (c) XRD patterns of Fe_3_O_4_, Fe_3_O_4_@dSiO_2_ and Fe_3_O_4_@dSiO_2_-(HE)_10_ nanospheres; (d) the magnetic hysteresis curves of Fe_3_O_4_, Fe_3_O_4_@dSiO_2_ and Fe_3_O_4_@dSiO_2_-(HE)_10_ at 300 K, and the inset shows pictures of the aqueous suspension of Fe_3_O_4_@dSiO_2_-(HE)_10_ (left) and the same suspension magnetically separated after 15 s (right).

The zeta potentials of Fe_3_O_4_@dSiO_2_, Fe_3_O_4_@dSiO_2_-SH and Fe_3_O_4_@dSiO_2_-(HE)_10_ were measured in binding solution (10 mM BR, pH 3.0), and their zeta potentials were +5.9, −0.5 and +17.5, respectively, as shown in Fig. 5a. The zeta potentials of Fe_3_O_4_@dSiO_2_-(HE)_10_ in different pH solutions indicated that the isoelectric point of Fe_3_O_4_@dSiO_2_-(HE)_10_ was 3.75 (Fig. 5b). The XRD pattern showed characteristic diffractograms of dSiO_2_, Fe_3_O_4_@dSiO_2_ and Fe_3_O_4_@dSiO_2_-(HE)_10_ (Fig. 5c). The 30.3°, 35.6°, 43.2°, 53.5°, 57.4° and 62.7° diffraction peaks of Fe_3_O_4_@dSiO_2_ at 2*θ* values were assigned to the crystal planes (220), (311), (400), (422), (511), and (440), respectively, which confirmed the successful synthesis of Fe_3_O_4_ with a cubic spinel structure (JCPDS# 19-0629). Fe_3_O_4_@dSiO_2_-(HE)_10_ showed a similar diffraction peak to Fe_3_O_4_@dSiO_2_, suggesting that Fe_3_O_4_@dSiO_2_-(HE)_10_ also had a typical magnetite structure. Moreover, the (HE)_10_ modification revealed no effect on the crystal structure of the functional unit. The broad diffraction peak near 22.7° was indexed to amorphous mesoporous SiO_2_. These diffraction peaks further confirmed that Fe_3_O_4_@dSiO_2_-(HE)_10_ was synthesized. Magnetic characteristics of Fe_3_O_4_, Fe_3_O_4_@dSiO_2_ and Fe_3_O_4_@dSiO_2_-(HE)_10_ at 300 K were investigated, and the magnetic hysteresis curves revealed their superparamagnetic features, as shown in Fig. 5d. The saturation magnetizations of Fe_3_O_4_, Fe_3_O_4_@dSiO_2_ and Fe_3_O_4_@dSiO_2_-(HE)_10_ were 39.47, 24.87 and 28.21 emu g^−1^, respectively, indicating a complete and quick magnetic separation of them from solution. The inset in Fig. 5d shows a very strong magnetic response of the prepared magnetic mesoporous silica beads. Typically, 15 s were long enough to completely collect the beads, suspended in pure water in a 1 × 1 cm cuvette. According to Fig. 5d, we have drawn normalized magnetic hysteresis curves of Fe_3_O_4_, Fe_3_O_4_@dSiO_2_ and Fe_3_O_4_@dSiO_2_-(HE)_10_ in Fig. S3.† As shown in Fig. S3,† the saturation magnetization of Fe_3_O_4_@dSiO_2_ was about 60% of that of Fe_3_O_4_, which indicated a decreased saturation magnetization of Fe_3_O_4_ after conjugation with dSiO_2_. Additionally, the (HE)_10_ modification had no effect on the saturation magnetization of Fe_3_O_4_@dSiO_2_.

### Application of Fe_3_O_4_@dSiO_2_-(HE)_10_

**Fig. 6:**
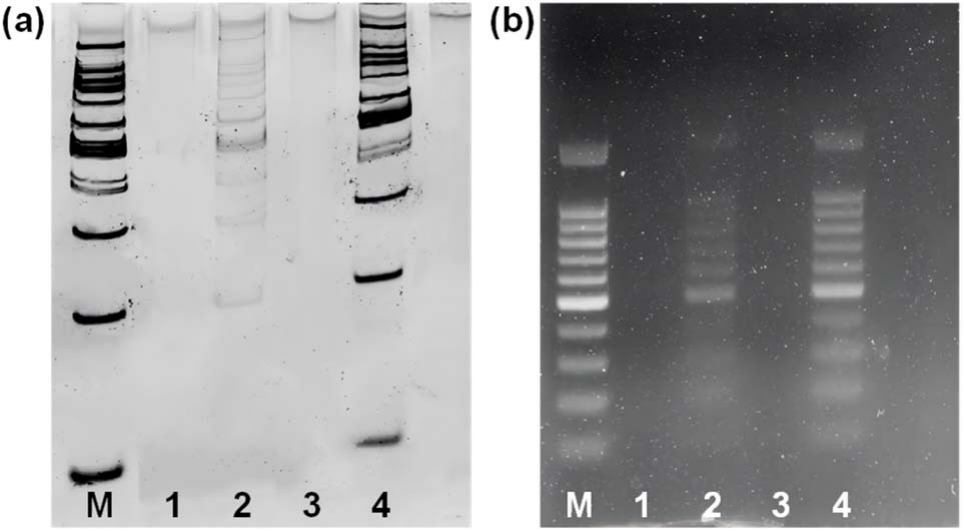
(a) 6% PAGE analysis; (b) 1% agarose gel electrophoresis of the DNA marker. Lane M: 100 bp DNA marker; lane 1: supernatant buffer (pH = 3.0) of sulfhydrated Fe_3_O_4_@dSiO_2_; lane 2: elution buffer of sulfhydrated Fe_3_O_4_@dSiO_2_; lane 3: supernatant buffer (pH = 8.0) of Fe_3_O_4_@dSiO_2_-(HE)_10_; lane 4: elution buffer of Fe_3_O_4_@dSiO_2_-(HE)_10_.

To investigate the potential of Fe_3_O_4_@dSiO_2_-(HE)_10_ for DNA extraction, a 100 bp DNA ladder marker was selected as a model to determine the recovery yields of DNA. The zeta potentials of Fe_3_O_4_@dSiO_2_-(HE)_10_ in different pH solutions (Fig. 5b) indicated that the isoelectric point of Fe_3_O_4_@dSiO_2_-(HE)_10_ was 3.75, and a certain amount of DNA can be captured by positively charged Fe_3_O_4_@dSiO_2_-(HE)_10_ in the pH range of 2.0–3.5. When the pH was above 3.75, the Fe_3_O_4_@dSiO_2_-(HE)_10_ and DNA were both negatively charged, leading to almost no DNA capture by Fe_3_O_4_@dSiO_2_-(HE)_10_ in the pH range of 4.0–10.0. Therefore, the solutions with pH = 3.0 and pH = 8.0 were selected as the binding solution for DNA capture and the elution solution for DNA release, respectively. As shown in PAGE analysis (Fig. 6a) and agarose gel electrophoresis (Fig. 6b), the lane of Fe_3_O_4_@dSiO_2_-(HE)_10_ was similar to that of the 100 bp DNA marker, while the lane of sulfhydrated Fe_3_O_4_@dSiO_2_ without modification of (HE)_10_ was different from that of the 100 bp DNA marker. The above results revealed that the obtained Fe_3_O_4_@dSiO_2_-(HE)_10_ magnetic beads could be used for DNA extraction based on the electrostatic interactions between Fe_3_O_4_@dSiO_2_-(HE)_10_ and DNA.

The DNA capture efficiency of Fe_3_O_4_@dSiO_2_-(HE)_10_ compared to that of Dynabeads MyOne Silane was investigated. As presented in agarose gel electrophoresis (Fig. S4†), the lane of Dynabeads MyOne Silane was similar to that of the 100 bp DNA marker, and the results were nearly the same those for Fe_3_O_4_@dSiO_2_-(HE)_10_, indicating the similar DNA capture efficiency of Fe_3_O_4_@dSiO_2_-(HE)_10_ and Dynabeads MyOne Silane. Finally, the potential applications of the obtained magnetic beads in DNA purifying for NGS library preparation were demonstrated, as described in Fig. S5.†

## Conclusions

In summary, monodisperse pH-sensitive oligopeptide magnetic mesoporous silica beads were successfully prepared *via* the thermolysis reaction of Fe(acac)_3_ by using large-pore dendritic silica colloids as templates. The ultra-large pore channels of dendritic silica colloids allowed the complete confinement of superparamagnetic iron oxide nanoparticles in the interior of the supports, enabling controlled dimension and monodispersity of the obtained magnetic beads. Due to the effective loading of superparamagnetic iron oxide nanoparticles, the as-prepared magnetic beads possessed an extremely high magnetic content of 59% and showed a strong magnetic response in magnetic fields. Furthermore, the magnetic mesoporous silica beads exhibited a pH-responsive behavior after modification with a highly pH-sensitive histidine-glutamate co-oligopeptide. All these remarkable features together with the relatively narrow dispersity of the resultant magnetic beads made them highly desirable for bioapplications, as demonstrated by deoxyribonucleic acid capture experiments.

## Experimental

### Reagents and instruments

Iron(iii) acetylacetonate (Fe(acac)_3_), cetyltrimethylammonium bromide (CTAB), sodium salicylate (NaSal), triethanolamine (TEA), and tetraethyl orthosilicate (TEOS) were purchased from Aladdin Chemical Reagent Co., Ltd. Hydrochloric acid (HCl), methanol, ethanol, (3-mercaptopropyl)trimethoxysilane (MPTMS), 2-pyrrolidone, and ammonia aqueous solution (25–28%) were received from Sinopharm Chemical Reagent Co., Ltd. Maleimide modified (HE)_10_ was obtained from Shanghai HongTide Biotechnology Co., Ltd. All chemicals were used as received without purification.

Zeta potentials and hydrodynamic diameters were measured using a Zetasizer Nano ZS dynamic light scattering particle size analyzer (Malvern, U.K.). Transmission electron microscopy (TEM) images were recorded using an FEI-Talos-S electron microscope operated at an accelerating voltage of 200 kV. The morphology of the magnetic beads was characterized using scanning electron microscopy (SEM, Regulus 8230). Magnetization curves of Fe_3_O_4_@dSiO_2_ and Fe_3_O_4_@dSiO_2_-(HE)_10_ were measured on a vibrating sample magnetometer (PPMS-1, Quantum Design, San Diego, USA) at 300 K. The magnetic content was determined using an inductively coupled plasma atomic emission spectrometer (Spectro, Arcos, Germany). Small-angle powder X-ray diffraction (XRD) testing was performed using a Rigaku D/max-2000 X-ray powder diffractometer (Rigaku) with Cu/Kα radiation of 1.5405 Å. Surface states of the magnetic mesoporous silica beads were determined by X-ray photoelectron spectroscopy (XPS, SUPRA).

### Preparation of Fe_3_O_4_

In a typical preparation of Fe_3_O_4_, a solution of 0.7063 g of Fe(acac)_3_ in 20 mL of 2-pyrrolidone was first purged with nitrogen to remove oxygen, and then heated to 245 °C and refluxed for 30 min. After cooling to room temperature, the product was collected by magnetic separation and washed several times with ethanol. The final black product was re-dispersed in 10 mL of ethanol.

### Synthesis of Fe_3_O_4_@dSiO_2_

The dendritic silica colloids (dSiO_2_) were synthesized according to a reported method with slight modifications. Briefly, 0.068 g of TEA was added to 25 mL of deionized water and stirred gently at 80 °C for 15 min. Then, 0.38 g of CTAB and 0.218 g of NaSal were added to the above solution, followed by stirring for another 15 min. Finally, 4 mL of TEOS was added into the water–TEA–CTAB–NaSal solution, which was stirred at 80 °C for another 3 h. The white product was collected by high-speed centrifugation (10 000 rpm) and washed several times with ethanol. The residual template was removed by HCl/methanol extraction at 60 °C for 12 h, and the purified product was re-dispersed in 30 mL of ethanol.

The typical synthesis of Fe_3_O_4_@dSiO_2_ was as follows: 20 mL of a 2-pyrrolidone solution containing 0.7063 g of Fe(acac)_3_ and 0.05 g of dSiO_2_ was purged with nitrogen for 30 min to remove oxygen. Then the reaction system was heated to 245 °C and refluxed for 30 min. After cooling to room temperature, the product was collected by magnetic separation and washed several times with ethanol. The final black product was re-dispersed in 10 mL of ethanol.

### Preparation of Fe_3_O_4_@dSiO_2_-(HE)_10_

10 mL of ethanol solution containing Fe_3_O_4_@dSiO_2_ was mixed with 56 μL of MPTMS and 250 μL of ammonia, and the mixture was stirred (600 rpm) at room temperature for 12 h. The obtained product was collected by magnetic separation and washed several times with ethanol. The obtained thiolated Fe_3_O_4_@dSiO_2_ (Fe_3_O_4_@dSiO_2_-SH) was re-dispersed in 10 mL of ethanol.

Conjugate addition reactions between thiolated Fe_3_O_4_@dSiO_2_ (0.05 g) and maleimide modified (HE)_10_ (0.001 g) were carried out in HEPES buffer (10 mM, pH 7.4). After being stirred for 30 min, the product was collected by magnetic separation and washed several times with ethanol/deionized water. The final product was re-dispersed in 1.0 mL of deionized water.

### DNA capture and elution assays with Fe_3_O_4_@dSiO_2_-(HE)_10_

To investigate the potential for DNA extraction from real samples, a 100 bp DNA ladder marker was selected as a model to determine the recovery yields of DNA using Fe_3_O_4_@dSiO_2_-(HE)_10_. The DNA ladder marker (1 μL) was dissolved in 1× TE buffer (9 μL, pH = 7.4) to prepare a DNA standard solution. The DNA standard solution, 25 μL of binding solution (10 nM BR buffer at pH = 3.0), and 2 μL of Fe_3_O_4_@dSiO_2_-(HE)_10_ suspension (30 mg mL^−1^) were mixed fully. Then the mixture was incubated at room temperature for 5 min. Afterwards, a magnetic stand was employed for magnetic separation and the supernatant was carefully removed. The DNA–Fe_3_O_4_@dSiO_2_-(HE)_10_ conjugates were washed twice with 100 μL of 80% (v/v) ethanol and allowed to dry at room temperature. Then the adsorbed DNA molecules were eluted from the conjugates by addition of 15 μL of elution solution (10 mM BR buffer at pH = 8.0) under vigorous shaking for 5 min. Finally, an appropriate amount of the supernatant was carefully taken for gel electrophoresis.

## Data availability

The data supporting this study are available within the article and its ESI.†

## Author contributions

Sihua Qian: formal analysis, writing – original draft, designed the study, performed experiments, analyzed the results, and wrote the manuscript. Yiting Wang: performed DNA capture and elution assays, analyzed the results, and wrote part of the manuscript. Junjie Fan: drew Fig. 3c, d, 4 and 5. Tong Kong: designed and drew Fig. 1. Yuhui Wang: provided valuable suggestions and discussed the results. Kaizhe Wang: provided valuable suggestions and discussed the results. Yufeng Liao: analyzed the results, conceived the idea and designed the study. Li Wang: conceived the idea and designed the study, writing – review & editing. Jianping Zheng: conceived the idea and designed the study, writing – review & editing.

## Conflicts of interest

There are no conflicts to declare.

## Supplementary Material

NA-OLF-D4NA00987H-s001
