# Supplementary material for: pH-Sensitive oligopeptide magnetic mesoporous silica beads for deoxyribonucleic acid extraction
Source: Nanoscale Adv. 2025 Jan 21;7(6):1663–70. doi: 10.1039/d4na00987h (PMC11780327; doi:10.1039/d4na00987h)
Supplement: NA-007-D4NA00987H-s001 [file NA-007-D4NA00987H-s001.pdf]

Supplementary Information (SI) for Nanoscale Advances.
This journal is © The Royal Society of Chemistry 2025

Supporting Information

pH-sensitive oligopeptide magnetic mesoporous silica beads for deoxyribonucleic acid extraction

Sihua Qian,[+b] Yiting Wang,[+a,b] Junjie Fan,[b] Tong Kong,[b] Yuhui Wang,[b] Kaizhe Wang,[b] Yufeng Liao,*[c] Li Wang*[a] and Jianping Zheng*[b]

[a] College of Chemistry, Jilin Normal University, Siping 136000, P. R. China.

[b] Ningbo Cixi institute of Biomedical Engineering, Laboratory of Advanced Theranostic Materials and Technology, Ningbo Institute of Materials Technology and Engineering, Chinese Academy of Sciences, Ningbo 315300, P. R. China.

[c] Department of Clinical Laboratory, Ningbo No. 2 Hospital, Ningbo 315010, P.R. China.

[+] These authors contributed equally to this work.

[*] Corresponding authors. E-mail: liaoyufengs@163.com; liwang@jlnu.edu.cn; zhengjianping@nimte.ac.cn

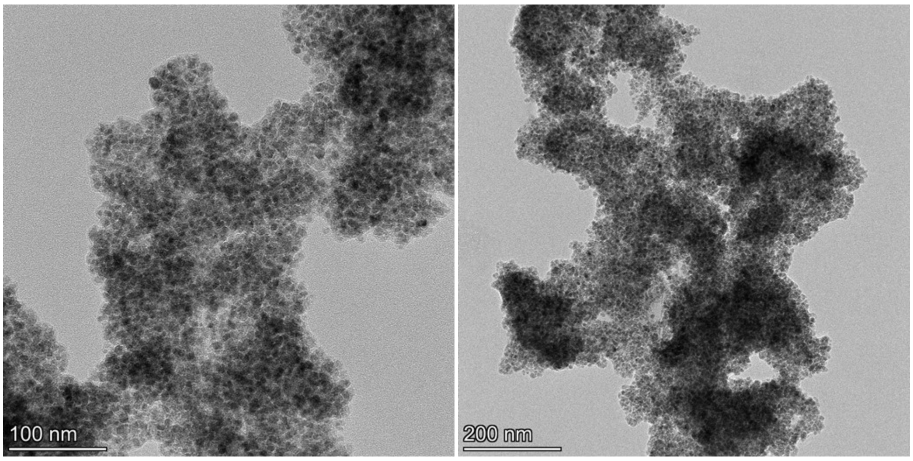

Fig. S1 TEM images of Fe_3O_4 nanoparticles; their average diameter was 5~10 nm; the scale bar corresponds to 100 nm (left) and 200 nm (right).

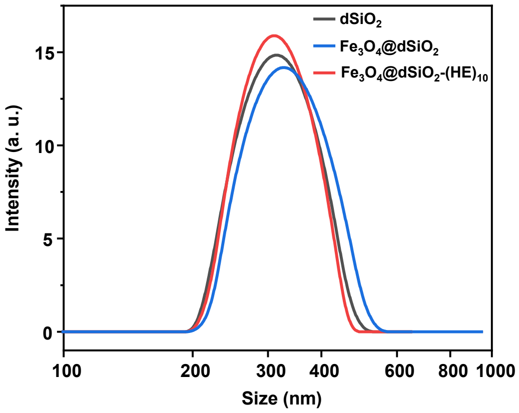

Fig. S2 Hydrodynamic diameters of $dSiO_2$, $Fe_3O_4@dSiO_2$ and $Fe_3O_4@dSiO_2$-(HE)$_{10}$ determined by DLS measurements.

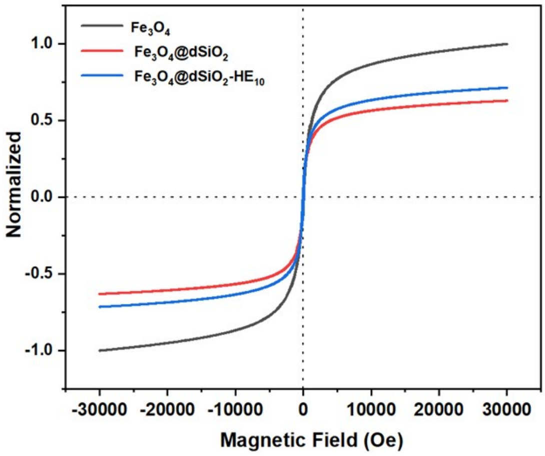

Fig. S3 The normalized magnetic hysteresis curves of Fe_3O_4, $Fe_3O_4@dSiO_2$ and $Fe_3O_4@dSiO_2$-(HE)$_{10}$ at 300 K.

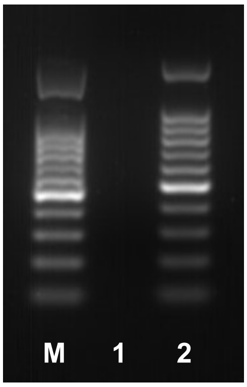

Fig. S4 1% Agarose gel electrophoresis of DNA marker. Lane M: 100 bp DNA marker; lane 1: Supernatant of Dynabeads MyOne Silane; lane 2: Elution of Dynabeads MyOne Silane.

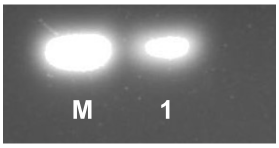

Fig. S5 Extraction of PCR amplification products from human genomic DNA. Lane M: Marker; Lane 1: Elution of Fe_3O_4@$dSiO_2$-$(HE)_{10}$.
